# Comparison of Bioavailability of Nitrates and Nitrites in 3 Species of *Amaranthus*: A Randomized, Double-Blind, Placebo-Controlled, 4-Way Crossover Study in Healthy Subjects Under Fasting Conditions

**DOI:** 10.1016/j.curtheres.2025.100789

**Published:** 2025-05-10

**Authors:** Deepa Suhag, Aparna Nilesh Kodre

**Affiliations:** 1 Amity Centre for Nanotechnology, Amity University Haryana, Gurugram, India; 2 Department of Medicine, Noble Hospital, Pune, India

**Keywords:** Amaranth, Bioequivalence, Dietary supplement, Nitrate, Nitrite, Pharmacokinetics

## Abstract

**Background:**

*Amaranthus* is a significant source of dietary nitrates, which have been known to improve aerobic capacity and exercise performance in physically active individuals. There is a significant data gap on nonpartitive pharmacokinetics and bioequivalence studies of nitrate and nitrite from 3 species of Amaranth (*A. hybridus, A. hypochondriacus*, and *A. tricolor*).

**Objective:**

This study aimed to assess the bioavailability of nitrates and nitrites from 3 *Amaranthus* species in a randomized, placebo-controlled design, thereby filling this gap.

**Methods:**

A double-blinded, 4-way crossover study was conducted in 16 healthy participants. Each participant enrolled in the study received a single dose of 2000 mg of *Amaranthus* extract or a placebo. The plasma and saliva samples were collected at specific intervals over 24 hours. Nitrate and nitrite concentrations were analyzed using a validated LCMS/MS method.

**Results:**

After the administration of amaranth extracts, both plasma and saliva samples were observed significantly higher levels of nitrate and nitrite compared with the placebo group. Pharmacokinetic variables (C_max_, AUC_0-t24_, and AUC0-∞) found a similar pattern for nitrite and nitrate in the 3 amaranth products but were significantly different from placebo (*P* < 0.05), in both plasma and saliva samples. Bioequivalence analysis confirmed significant bioequivalence among the 3 amaranth extracts for nitrite and nitrate.

**Conclusions:**

This study concludes that the 3 species of *Amaranthus*—*A. hybridus, A. hypochondriacus*, and *A. tricolor* are bioequivalent in terms of plasma and saliva nitrate and nitrite levels from a single dose of 2000 mg amaranth extract and have higher bioavailability than placebo. These findings report that *Amaranthus* extracts could potentially act as a daily diet supplement for improving the cardiovascular and neurogenerative health of the body, particularly aging people.

## Introduction

Recently, there has been an increased interest in the potential health benefits of dietary nitrates and nitrites, especially for cardiovascular health and cognitive function. Nitrates and nitrites are the fundamental compounds naturally found mainly in green vegetables.^1^^,^^2^ *Amaranthus* refers to a group of leafy green vegetables that are particularly high in nitrates and known for their health-beneficial properties.^3^ Nitrate and nitrite supplementation was shown in multiple studies to produce beneficial effects on cardiovascular function by enhancing nitric oxide (NO) bioavailability, which leads to improved endothelial function, lowered blood pressure, and increased performance during exercise.^4^, ^5^, ^6^ Enhanced NO bioavailability improves the endothelial function, which contributes to overall cardiovascular health.^7^ A recent study reported reduced microvascular inflammation, endothelial dysfunction, and c-reactive protein in animals administered with a high-cholesterol diet.^8^^,^^9^

Additionally, emerging evidence suggests a potential role for NO in cognitive function, with NO playing a crucial role in regulating cerebral blood flow and neurotransmission.^10^ NO has been implicated in modulating synaptic plasticity, neuronal survival, and cognitive performance.^11^ Therefore, interventions that enhance NO bioavailability may have implications for cognitive function and neuroprotection. Despite the growing interest in the potential health benefits of dietary nitrates and nitrites, there remains a need for further research to elucidate their bioavailability and effects on human health. Furthermore, the bioavailability of nitrates and nitrites may vary depending on the source and formulation of the dietary supplement. Leafy vegetables, roots, and rhizomes of certain edible plants are reportedly major sources of dietary nitrate. Amaranth, a leafy vegetable generally included in the daily diet in tropical countries such as Africa, India, Sri Lanka, and Bangladesh reported to have a large amount of nitrate along with carotenoids, iron, calcium, ascorbic acid, and proteins.^12^^,^^13^ Although the inclusion of these leafy vegetables in large quantities could provide dietary nitrates for the human body, it is not enough to generate significant levels of nitrate and nitrite in blood to produce a clinically significant impact. Separate supplementation of nitrate found clinically significant increase in nitrate and nitrite in the body.^14^

Although previous studies have reported the bioavailability of nitrate and nitrite from different dietary sources, there are limited data availability on the comparative assessment of pharmacokinetics and bioequivalence of different *Amaranthus* species-based formulations against placebo. The present study aims to fill this gap by assessing the bioavailability of nitrates and nitrites from 3 different species of amaranth (*A. hybridus, A. hypochondriacus*, and *A. tricolor*) in healthy adult human subjects under fasting conditions. The study design employs a randomized placebo-controlled, double-blind, double-sequential 4-way crossover design to standardly assess the bioavailability of nitrates and nitrites from all the formulations.

## Materials and Methods

### Study design

**Figure 1:**
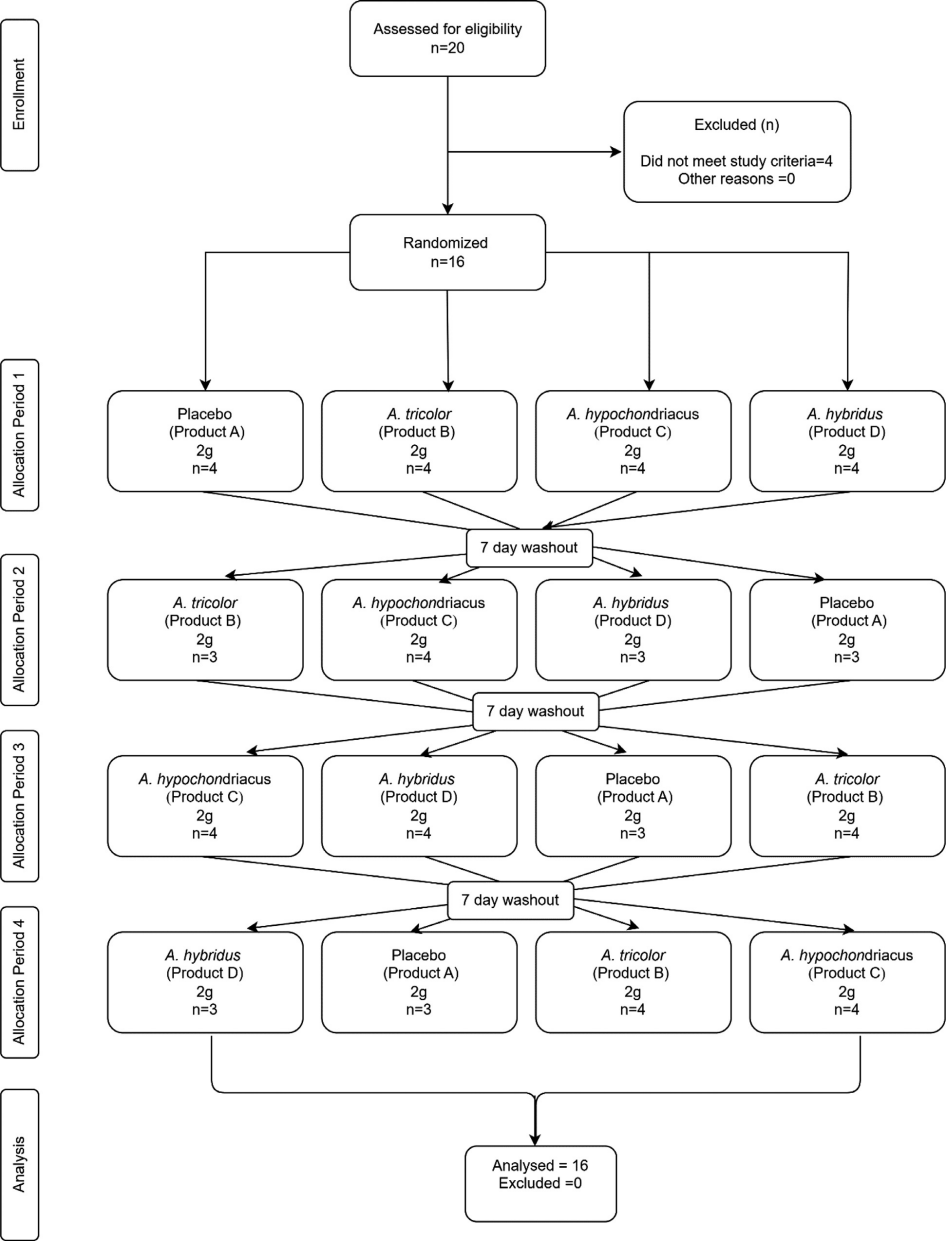
Consort flow chart showing the flow of participants through each stage of the randomized crossover trial.

This was a 4-period, 4-way crossover randomized double-blinded placebo-controlled study conducted on healthy adult participants in a single center under fasting conditions. The study aimed to assess the comparative oral bioavailability of nitrates and nitrites in plasma and saliva upon a dosage of 2000 mg of *Amaranthus* extracts taken from 3 different species. The randomized, crossover trial design adopted in this study minimizes intersubject variability by allowing each participant to serve as their own control,^15^ which enhances statistical power by reducing the effect of individual differences in nitrate metabolism, thereby requiring a smaller sample size to detect significant changes. Furthermore, a crossover design allows for direct within-subject comparison of bioavailability across different interventions, making the bioequivalence evaluation more precise.^16^ The subjects were housed in the clinical facility of the Clinical Pharmacology and Pharmacokinetic Unit, Noble Hospital, Pune, from at least 12 hours before the investigational product (IP) administration to at least 24 hours after the IP administration. The IP was administered according to a preprepared allocation concealed randomization list. The washout window was at least 7 days between each dosing of the study (Figure 1).

### Subjects and screening

A total of 20 healthy volunteers were screened, out of which 4 subjects did not meet the study criteria, and hence, a total of 16 subjects were enrolled for the study. Subjects were screened within 21 days before dosing and included demographic assessment, vital signs, medical history, ECG, chest X-ray, and clinical laboratory tests (hematology, biochemistry, serology, and urine analysis). Clinical laboratory tests, ECG, and chest X-ray assessments were conducted to ensure that there were no underlying conditions that could potentially affect the study outcomes, and hence ensure that only healthy individuals free from cardiovascular, metabolic, renal, hepatic, or respiratory disorders were included in the study.

#### Inclusion criteria

Participants were considered eligible to be included in the study if they were aged between 18 and 55 years and were in good general health. The participants with a body mass index between 18.5 and 25 kg m^2^. Participants to be included need to have no history of cardiovascular, metabolic, renal, or hepatic disorders. Enrolled participants are expected to be nonsmokers and not use tobacco products. Subjects were required not to consume xanthine-containing food or beverages within 24 hours before the dosing of each period. Participants who had not consumed nitrate-rich diets in the past 72 hours were included. Included participants are also expected to be willing to abstain from alcohol, caffeine, and grapefruit for 48–72 hours before dosing.

#### Exclusion criteria

Participants were excluded if they had a history of cardiovascular, metabolic, renal, or hepatic diseases. Pregnant or lactating participants were excluded. Smokers or participants who had used tobacco in the past 6 months, history of substance abuse, or excessive alcohol consumption were excluded. Participants who had used nitrate-based medications or enzyme-modifying drugs in the last 4 weeks or had allergies to plant extracts or related compounds were also excluded.

### Ethical conduct of the study

This study was conducted in accordance with the current version of the declaration of Helsinki (52nd WMA General Assembly, Edinburgh, Scotland, October 2000) and agreement with the International Conference on Harmonisation guidelines on Good Clinical Practise, with approval from the Institutional Ethics Committee, Noble Hospital, Pune, India, dated 6 April 2018. This study was registered in CTRI with number CTRI/2018/06/014440 [Registered on: 06/06/2018]. The study was conducted at Noble Hospital, Pune, from August 1, 2018 to August 29, 2018. All subjects were provided with information sheet and given sufficient time to read and understand the information before voluntarily signing the informed consent, after explaining them all procedures involved in this study (aims, methodology, potential risks, and anticipated benefits).

### Intervention

The study utilized water extracts of *A. tricolor* (Product B, Batch No: OB-M2/18-19), *Amaranthus hypochondriacus* (Product C, Batch No: OB-M3/18-19), and *Amaranthus hybridus* (Product D, Batch No: OB-M4/18-19), which were standardized to a minimum of 9% nitrate. The placebo was formulated using maltodextrin with caramel color (Product A, Batch No: OB-M1/18-19). These extracts and placebo were procured from Arjuna Natural Pvt. Ltd. The interventions were packed in labeled tight screw cap vials having 2 g extracts/ placebo in powder form. The powder was emptied from the vials into a glass containing 240-mL lukewarm water, stirred for 1 minute, and was instructed to consume it immediately, reducing chances of detecting any significant taste variations. An additional 50 mL rinse ensured complete intake of the powder. Subjects received their doses at scheduled times and remained in a semirecumbent position for the initial 4 hours after administration. Afterward, they were allowed to move around but were advised against strenuous physical activity. The order of administration for each subject followed the randomization schedule prepared by the biostatistician, ensuring balanced randomization with controlled access to the code.

### Sample collection

Blood samples of the subjects were withdrawn by an indwelling cannula placed in a forearm vein or by direct vein puncture using a disposable sterilized syringe in cases of clotting of cannula. Samples were collected through the IV cannula for the first 12 hours, and subsequent samples after 12 hours were drawn by sterile direct venipuncture, because of the increased risk of cannula clotting or loss of patency, which could compromise sample integrity. The predose blood samples were collected within 30 minutes before dosing, and postdose in-house blood samples were collected within ±2 minutes of the scheduled time. The actual time of sample collection was taken into consideration for pharmacokinetic calculation. Intravenous indwelling cannula was kept in situ as long as possible by injecting 0.5 mL of 5 IU/mL of heparin in normal saline solution to maintain the cannula patent. While sampling through a cannula, blood samples were collected after discarding the first 0.5 mL of heparinized blood from the cannula. The blood samples were collected in prelabeled (label mentioning study no., subject no., period no., and sampling time point) Lithium heparin plastic blood collecting tubes. The collection schedule for plasma and saliva samples is summarized in Table 1.

**Table 1:** Collection schedule for plasma and saliva samples.

Sample type	Predose (0 h)	Postdose time points (h)
Plasma	Yes	0.15, 0.5, 0.75, 1.0, 1.5, 2.0, 2.5, 3.0, 3.5, 4.0, 5.0, 6.0, 9.0, 12.0, 16.0, 24.0
Saliva	Yes	0.15, 0.5, 0.75, 1.0, 1.5, 2.0, 2.5, 3.0, 3.5, 4.0, 5.0, 6.0,8.0,24.0

After collection, blood samples were immediately placed in an ice bath before being transferred to a refrigerated centrifuge within 30 minutes. The samples were then spun at 5000 ± 100 rpm at 4°C for 15 minutes. Unstimulated whole saliva was collected by passive drooling into a sterile polypropylene tube at each time point as detailed in Table 1. Participants were instructed not to swallow or talk 1 minute before sample collection to reduce variability in nitrate/nitrate concentrations. Saliva samples were promptly chilled after collection and then transferred to a −80°C deep freezer for storage. All plasma and saliva samples were stored at −80°C ± 10°C at the clinical facility until shipment to the analytical facility, where they were maintained at the same temperature until analysis to prevent degradation. Sample stability was ensured by analyzing all specimens within 30 days of collection.

After the clinical phase, plasma samples were transferred to the R&D laboratory of Arjuna Natural Pvt. Ltd, under cold conditions (−80°C). Each sample (plasma and saliva) was adequately labeled with the subject initial, randomization no, period of study, time point, and aliquot number. While transferring the samples, adequate measures were taken for maintaining temperature (−80°) and integrity of samples.

### Randomization, blinding, and unblinding

The randomization schedule was generated using WinPepi version 11.65 software using balanced block randomization. The investigator and the participants were blinded to the intervention. The identity of the IPs was concealed using an alphanumeric code. Sealed in opaque envelopes containing the identity of the IP was constituted and kept under the restricted access of the pharmacist to be used for emergency unblinding.

### Bioanalytical methods

Plasma and saliva samples were analyzed for nitrate (NO₃ˉ) and nitrite (NO₂ˉ) using a validated Ultra Performance Liquid Chromatography (UPLC) method with an ACQUITY UPLC BEH C18 column and Waters Empower 2 software. Processed samples were filtered (0.2 µm) and injected for NO₃ˉ quantification at 222 nm, whereas NO₂ˉ was derivatized with Griess reagent and detected at 520 nm. Samples exceeding the assay range were diluted and reassayed, whereas those below the lower limit of quantitation were reported as below the lower limit of quantitation. Limit of Detection (LOD) and Limit of Quantification (LOQ) for NO₃ˉ were 0.01 µg/mL and 0.05 µg/mL, respectively. Because of contamination in blanks, LOD for NO₂ˉ was undetermined, but LOQ was 5 ng/mL. Intraday (6 times/day) and interday (over 3 days) tests reported high precision, with %CV below 2.1%.

### Pharmacokinetic analysis

Data from all subjects were included in the pharmacokinetic analysis and were analyzed in the “*pkr*” package in the R 4.0 statistical tool by noncompartmental methods. When performing NCA calculations, Lambda Z was used instead of K_el_ because the supplement follows a simple one-compartment model with a single exponential elimination phase.

All concentration values below the lower limit of quantitation were set to “zero.” The missing data were handled using the last observation carried forward strategy. The risk of spurious data was minimized by implementing data quality checks in data collection, recording, and analysis, using standardized systems for data collection, implementing robust data security measures, and ensuring proper data storage and access controls.

### Statistical analysis

Statistical analyses of nitrate/nitrite concentrations in plasma/saliva were performed using the R programming tool and SPSS version 20.0. The mean, standard deviation, and 95% CI for each pharmacokinetic parameter (C_max_, AUC_0-t_, AUC_0-∞_, and T_max_) were obtained according to period and treatment. Log-transformed pharmacokinetic parameters (C_max_, AUC_0-t_, and AUC_0-∞_) were analyzed using a general linear modeling approach with treatment and period as main effects. The least squares mean was obtained for each level of the fixed effects. The paired analysis was performed for each parameter to test the statistical significance of difference between treatments as well as periods using Tukey post hoc analysis. The 90% CI was obtained for the mean differences of the log-transformed variables and compared with (−20%, −25%) limits to decide about bioequivalence. The predefined bioequivalence margin was 80%–125%, in accordance with the U.S. Food and Drug Administration and the European Medicines Agency guidelines for bioequivalence studies.^17^

## Results

Sixteen healthy human subjects between the age group 18–55 years were enrolled in the study. In the first period, all 16 subjects consumed the dosage, and 24 hours blood and saliva samples were collected from them. One subject was lost to follow-up from the second period onward, one subject was missing for periods 2 and 4, and another was missing for only period 2. There was no protocol violation or any sample withdrawals during the different stages of the study.

### Analysis of plasma nitrate

**Figure 2:**
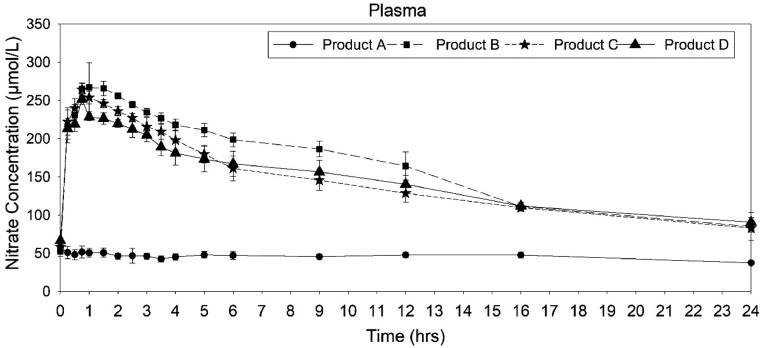
Mean nitrate plasma concentration−time curve after administration of placebo and *Amaranthus* products.

The mean plasma concentration−time profiles for nitrate found similar profiles for *Amaranthus hybridus* extract (product B), *A. hypochondriacus* (product C), *A. tricolor* extract (product D), whereas the plasma nitrate concentration profile of placebo (product A) was dissimilar to the *Amaranthus* products (Figure 2). The descriptive pharmacokinetic parameters are given in Table 2. The mean C_max_ of placebo (62.81 ± 7.03 ng/mL) was lower than products B, C, and D (275.19 ± 7.013, 265.59 ± 6.96, and 252.348 ± 4.16 ng/mL, respectively); however, among B, C, and D, there was no significant difference. The half-life was higher for placebo compared with B, C, and D, and that was because the blood always has a nitrate steady state, which shows up in the concentration plot without a dip, resulting in a higher half-life.

**Table 2:** Descriptive statistics for pharmacokinetic variables.

		Plasma nitrate			Plasma nitrite			Saliva nitrate			Saliva nitrite		
			95% CI			95% CI			95% CI			95% CI	
		Mean ± SD	Lower bound	Upper bound	Mean ± SD	Lower bound	Upper bound	Mean ± SD	Lower bound	Upper bound	Mean ± SD	Lower bound	Upper bound
Lambda z	A	0.0202 ± 0.01	0.0175	0.0229	0.0231 ± 0.02	0.0109	0.0353	0.0633 ± 0.01	0.0554	0.0713	0.0106 ± 0.01	0.0061	0.0151
	B	0.0544 ± 0.02	0.0444	0.0645	0.0136 ± 0	0.0132	0.014	0.0801 ± 0.02	0.0691	0.091	0.066 ± 0.02	0.0572	0.0748
	C	0.0373 ± 0.01	0.0326	0.042	0.0138 ± 0	0.0134	0.0142	0.0947 ± 0.02	0.0831	0.1063	0.0624 ± 0.01	0.0552	0.0696
	D	0.037 ± 0	0.0355	0.0386	0.0143 ± 0	0.0138	0.0147	0.0912 ± 0.03	0.0775	0.1049	0.0604 ± 0.01	0.0532	0.0675
t_half	A	36.4212 ± 9.29	31.4691	41.3734	92.6632 ± 151.73	11.8114	173.515	11.9022 ± 4.66	9.4192	14.3852	115.9781 ± 98.52	63.4806	168.4756
	B	13.7363 ± 3.11	12.0773	15.3953	51.0327 ± 2.8	49.5422	52.5233	9.4283 ± 3.5	7.564	11.2926	11.1343 ± 2.73	9.6821	12.5865
	C	19.3681 ± 3.55	17.474	21.2621	50.4152 ± 2.64	49.0063	51.8241	7.7192 ± 1.89	6.7108	8.7276	11.62 ± 2.56	10.2557	12.9843
	D	18.8392 ± 1.7	17.9332	19.7452	48.7985 ± 2.92	47.2444	50.3525	8.4039 ± 3.14	6.7283	10.0795	12.0671 ± 2.88	10.5333	13.6009
Tmax	A	1 ± 1.88	−0.0016	2.0016	4.3125 ± 3.66	2.3622	6.2628	0.7188 ± 0.86	0.2628	1.1747	2.0625 ± 1.48	1.2729	2.8521
	B	0.9219 ± 0.2	0.8162	1.0275	0.7656 ± 0.06	0.7323	0.7989	0.8438 ± 0.2	0.7363	0.9512	0.7969 ± 0.14	0.7244	0.8693
	C	0.875 ± 0.57	0.5712	1.1788	0.7969 ± 0.14	0.7244	0.8693	1 ± 0.46	0.7568	1.2432	0.9375 ± 0.57	0.6357	1.2393
	D	0.8594 ± 0.44	0.6262	1.0925	0.8281 ± 0.18	0.7343	0.9219	0.8594 ± 0.2	0.7509	0.9678	0.7969 ± 0.14	0.7244	0.8693
Cmax	A	62.8088 ± 7.03	59.0628	66.5547	0.5638 ± 0.08	0.5187	0.6088	766.23 ± 142.7	690.1899	842.2701	262.3519 ± 10.49	256.7644	267.9394
	B	275.1934 ± 7.01	271.4563	278.9305	1.7331 ± 0.01	1.7274	1.7389	3472.556 ± 655.38	3123.329	3821.783	1520.9 ± 302.6	1359.653	1682.147
	C	265.5909 ± 6.96	261.884	269.2978	1.7206 ± 0.02	1.7099	1.7314	3845.335 ± 364.88	3650.903	4039.767	1681.2013 ± 282.72	1530.552	1831.851
	D	252.3483 ± 4.16	250.1301	254.5664	1.7206 ± 0.02	1.7092	1.732	3710.8725 ± 410.94	3491.899	3929.846	1585.8281 ± 287.05	1432.872	1738.784
AUC0_t	A	1094.4795 ± 42.32	1071.93	1117.029	8.4426 ± 1.22	7.7942	9.0909	6972.12 ± 1462.61	6192.755	7751.491	4459.4248 ± 400.55	4245.986	4672.864
	B	3811.5047 ± 135.08	3739.525	3883.485	13.9415 ± 0.09	13.8939	13.989	19343.26 ± 4116.99	17149.47	21537.05	10839.65 ± 1681.14	9943.839	11735.47
	C	3378.9821 ± 159.13	3294.189	3463.776	13.9904 ± 0.14	13.9139	14.0669	22490.02 ± 2836.3	20978.66	24001.38	11059.41 ± 1652.21	10179.01	11939.81
	D	3427.4061 ± 192.61	3324.769	3530.043	14.0128 ± 0.16	13.9272	14.0984	21541.11 ± 3355.93	19752.86	23329.36	11009.70 ± 1681.53	10113.68	11905.73
AUC0_inf	A	3067.9971 ± 547.3	2776.361	3359.634	54.5603 ± 77.15	13.451	95.6697	8806.906 ± 961.36	8294.635	9319.177	35760.83 ± 28991.8	20312.19	51209.47
	B	5568.1488 ± 695.94	5197.311	5938.987	39.9383 ± 1.46	39.1598	40.7168	22990.91 ± 4975.23	20339.8	25642.02	13649.08 ± 2106.03	12526.86	14771.31
	C	5712.2795 ± 424.06	5486.315	5938.244	39.7451 ± 0.98	39.2238	40.2664	25592.44 ± 2944.76	24023.29	27161.6	14086.75 ± 2100.89	12967.26	15206.23
	D	5876.4034 ± 213.56	5762.607	5990.199	39.0287 ± 1.21	38.3831	39.6743	25217.17 ± 4186.66	22986.25	27448.08	14218.65 ± 2297.98	12994.15	15443.16
MRT	A	53.4844 ± 12.71	46.7118	60.257	136.4314 ± 217.83	20.3603	252.5026	15.4911 ± 6.57	11.9881	18.9941	168.4671 ± 142.02	92.7909	244.1432
	B	20.3767 ± 4.17	18.1555	22.5979	66.6635 ± 3.73	64.6739	68.6531	11.4816 ± 3.51	9.6121	13.3511	14.3537 ± 3.43	12.5263	16.1812
	C	27.0966 ± 4.53	24.6821	29.511	65.8404 ± 3.35	64.0544	67.6265	9.7292 ± 1.92	8.7049	10.7535	14.7833 ± 2.94	13.2146	16.352
	D	27.2405 ± 1.96	26.1941	28.2869	63.7273 ± 3.74	61.7333	65.7212	10.7774 ± 2.99	9.1827	12.3721	15.3428 ± 3.39	13.5357	17.1499
Vz	A	33.854 ± 3.17	32.1635	35.5446	4182.91 ± 1091.64	3601.213	4764.605	4.0577 ± 2.05	2.9642	5.1513	9.1334 ± 0.92	8.6434	9.6234
	B	7.0212 ± 0.95	6.517	7.5254	3685.012 ± 89.99	3637.062	3732.962	1.1945 ± 0.35	1.01	1.3791	2.3756 ± 0.56	2.0772	2.6739
	C	9.7124 ± 1.25	9.0475	10.3774	3658.207 ± 124.49	3591.869	3724.545	0.8787 ± 0.22	0.761	0.9965	2.4067 ± 0.52	2.1288	2.6847
	D	9.2784 ± 1.13	8.6755	9.8813	3605.61 ± 134.76	3533.803	3677.419	0.9714 ± 0.34	0.7912	1.1515	2.4729 ± 0.53	2.1895	2.7564
Cl	A	0.67 ± 0.11	0.6111	0.7289	76.8745 ± 55.36	47.373	106.376	0.2297 ± 0.03	0.2161	0.2432	0.0928 ± 0.07	0.0572	0.1284
	B	0.3658 ± 0.06	0.3356	0.396	50.141 ± 1.86	49.1489	51.1332	0.091 ± 0.02	0.0803	0.1017	0.1499 ± 0.02	0.1371	0.1628
	C	0.3522 ± 0.03	0.3363	0.3681	50.3501 ± 1.28	49.6705	51.0297	0.0792 ± 0.01	0.074	0.0844	0.1449 ± 0.02	0.1336	0.1563
	D	0.3408 ± 0.01	0.3338	0.3478	51.291 ± 1.61	50.4355	52.1465	0.0819 ± 0.02	0.0728	0.091	0.144 ± 0.02	0.1321	0.1558

The linear model and pairwise comparison outcomes of log-transformed Cmax, AUC0-t, and AUC0-∞, when compared within the (−20%, 25%) range, indicated bioequivalence among the *Amaranthus* products A, B, and C. Conversely, the Cmax, AUC0-t, and AUC0-∞ values observed for the placebo did not demonstrate bioequivalence with the *Amaranthus* products for plasma nitrate (Table 3). The bioavailability of *Amaranthus* products A, B, and C is notably 3.5, 3, and 3.1 times higher, respectively, than that of the placebo.

**Table 3:** Bioequivalence assessment of different products for plasma nitrate.

Dependent variable			Mean difference (I–J)	SE	Sig.	95% CI		95% CI (Transformed)		
						Lower bound	Upper bound	Lower bound	Upper bound	Bioequivalence
C_max_	A	B	−0.64385*	0.00872	0.000	−0.6669	−0.6208	−48.67	−46.2494	False
		C	−0.62842*	0.00872	0.000	−0.6515	−0.6054	−47.8716	−45.4134	False
		D	−0.60630*	0.00872	0.000	−0.6293	−0.5833	−46.7054	−44.1921	False
	B	C	0.01543	0.00872	0.298	−0.0076	0.0385	−0.75769	3.922316	True
		D	0.03756*	0.00872	0.000	0.0145	0.0606	1.462607	6.247317	True
	C	D	0.02213	0.00872	0.064	−0.0009	0.0452	−0.09133	4.6201	True
AUC0_t	A	B	−0.54193*	0.00712	0.000	−0.5607	−0.5231	−42.9217	−40.7325	False
		C	−0.48945*	0.00712	0.000	−0.5083	−0.4706	−39.8466	−37.5394	False
		D	−0.49533*	0.00712	0.000	−0.5141	−0.4765	−40.199	−37.9053	False
	B	C	0.05247*	0.00712	0.000	0.0337	0.0713	3.422799	7.389599	True
		D	0.04660*	0.00712	0.000	0.0278	0.0654	2.81694	6.760501	True
	C	D	−0.00588	0.00712	0.842	−0.0247	0.0129	−2.43919	1.302777	True
AUC0_inf	A	B	−0.26129*	0.01824	0.000	−0.3095	−0.2131	−26.6185	−19.191	False
		C	−0.27484*	0.01824	0.000	−0.3231	−0.2266	−27.6064	−20.2788	False
		D	−0.28809*	0.01824	0.000	−0.3363	−0.2399	−28.5593	−21.3281	False
	B	C	−0.01355	0.01824	0.879	−0.0618	0.0347	−5.98941	3.526224	True
		D	−0.02680	0.01824	0.462	−0.0750	0.0214	−7.22675	2.163632	True
	C	D	−0.01325	0.01824	0.886	−0.0615	0.0350	−5.96075	3.557781	True

“*” represents statistically significant mean difference. Bioequivalence margins were set at 80%–125%.

### Analysis of plasma nitrite

**Figure 3:**
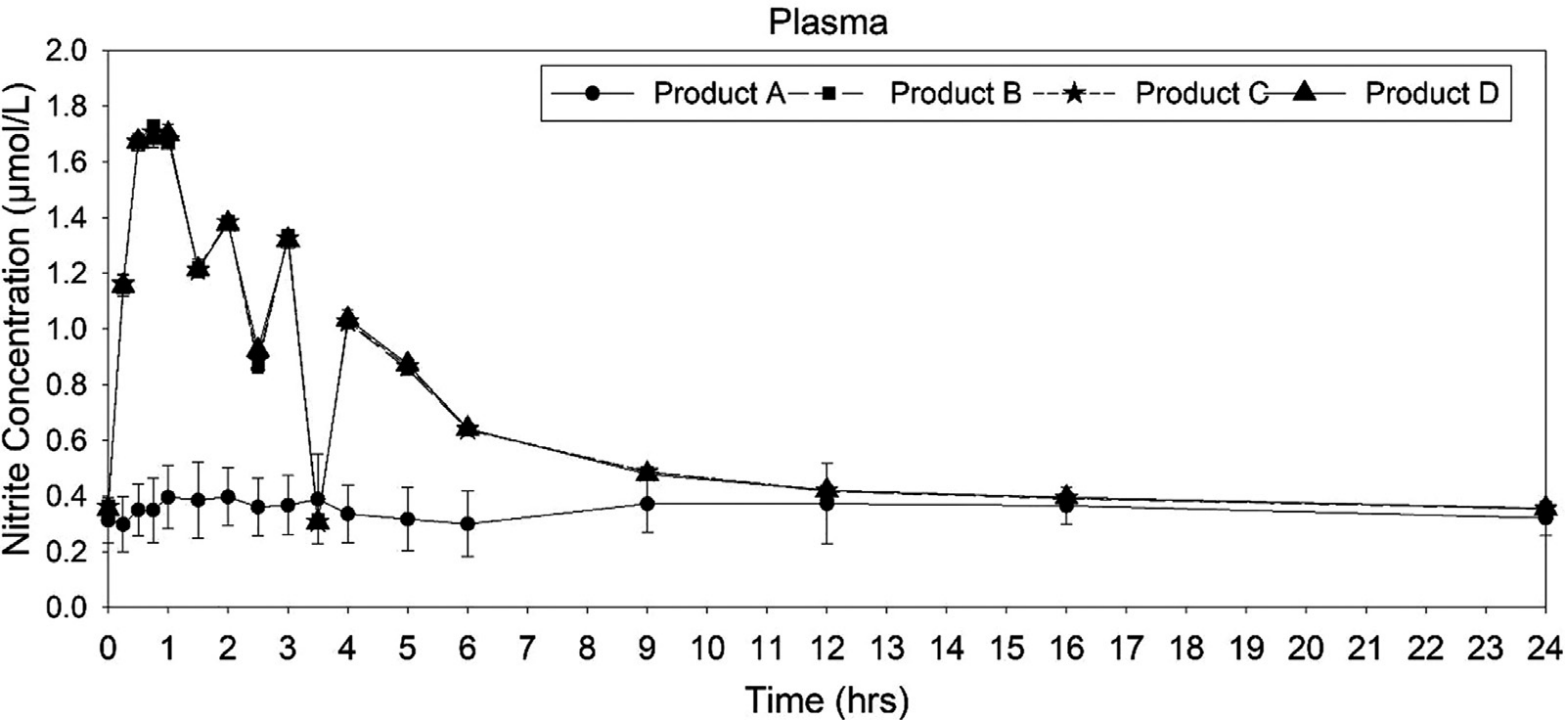
Mean nitrite plasma concentration−time curve after administration of placebo and *Amaranthus* products.

The mean plasma concentration−time profiles for nitrite found similar profiles for the *Amaranthus* products (products B, C, and D), whereas the plasma nitrite concentration profile of placebo (product A) was dissimilar to the *Amaranthus* products (Figure 3). The descriptive pharmacokinetic parameters are given in Table 2. The mean C_max_ of placebo (0.56 ± 0.08 ng/mL) was lesser than that of *Amaranthus*. The mean C_max_ of product B (1.73 ± 0.01 ng/mL), product C (1.72 ± 0.02 ng/mL), and product D (1.72 ± 0.02 ng/mL) was found to be similar. The mean T_max_ was found comparable for the *Amaranthus* administration (0.77 ± 0.06 hours, 0.796 ± 0.14 hours, and 0.83 ± 0.18 hours for products B, C, and D, respectively). T_max_ obtained from the placebo administration (4.31 ± 3.66 hours) showed a statistically significant difference with amaranth extracts. Placebo administration showed a higher elimination half-life (92.66 ± 151.73 hours), compared with the *Amaranthus* administrations with the elimination half-life of 51.03 ± 2.797 hours, 50.42 ± 2.64 hours, and 48.798 ± 2.92 hours for products B, C, and D, respectively.

The linear model and pairwise comparison outcomes of log-transformed Cmax, AUC0-t, and AUC0-∞, when compared within the (−20%, 25%) range, indicated bioequivalence among the *Amaranthus* products A, B, and C. Conversely, the Cmax, AUC0-t, and AUC0-∞ values observed for the placebo did not demonstrate bioequivalence with the *Amaranthus* products for plasma nitrite (Table 4). The bioavailability of *Amaranthus* products A, B, and C is notably 2.7, 3.2, and 3.1 times higher, respectively, than that of the placebo.

**Table 4:** Boequivalence assessment of different products for plasma nitrite.

Dependent variable			Mean difference (I–J)	SE	Sig.	95% CI		95% CI (Transformed)		
						Lower bound	Upper bound	Lower bound	Upper bound	Bioequivalence
Cmax	A	B	−0.49232*	0.01162	0	−0.523	−0.4616	−40.73	−36.97	False
		C	−0.48916*	0.01162	0	−0.5199	−0.4585	−40.54	−36.77	False
		D	−0.48916*	0.01162	0	−0.5199	−0.4585	−40.54	−36.77	False
	B	C	0.00316	0.01162	0.993	−0.0275	0.0339	−2.72	3.44	True
		D	0.00317	0.01162	0.993	−0.0275	0.0339	−2.72	3.44	True
	C	D	0	0.01162	1	−0.0307	0.0307	−3.02	3.12	True
AUC0_t	A	B	−0.22225*	0.01152	0	−0.2527	−0.1918	−22.33	−17.45	False
		C	−0.22375*	0.01152	0	−0.2542	−0.1933	−22.45	−17.58	False
		D	−0.22444*	0.01152	0	−0.2549	−0.194	−22.50	−17.63	False
	B	C	−0.00151	0.01152	0.999	−0.032	0.0289	−3.14	2.94	True
		D	−0.0022	0.01152	0.998	−0.0326	0.0282	−3.21	2.86	True
	C	D	−0.00069	0.01152	1	−0.0311	0.0298	−3.07	3.02	True
AUC0_inf	A	B	−0.06188	0.06708	0.793	−0.2391	0.1154	−21.27	12.23	False
		C	−0.05992	0.06708	0.808	−0.2372	0.1173	−21.12	12.45	False
		D	−0.05195	0.06708	0.866	−0.2292	0.1253	−20.48	13.35	False
	B	C	0.00196	0.06708	1	−0.1753	0.1792	−16.08	19.63	True
		D	0.00993	0.06708	0.999	−0.1673	0.1872	−15.41	20.59	True
	C	D	0.00797	0.06708	0.999	−0.1693	0.1852	−15.57	20.35	True

“*” represents statistically significant mean difference. Bioequivalence margins were set at 80%–125%.

### Analysis of saliva nitrate

**Figure 4:**
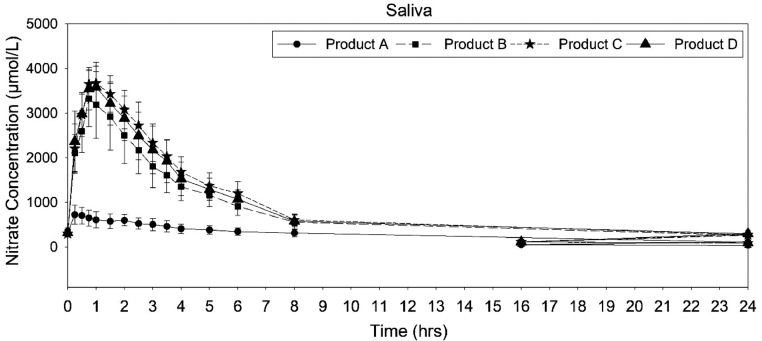
Mean nitrate saliva concentration−time curve after administration of placebo and *Amaranthus* products.

The mean saliva concentration−time profiles for nitrate found similar profiles for the *Amaranthus* products (products B, C, and D) but not compared with placebo (Figure 4). The descriptive pharmacokinetic parameters are given in Table 2. The mean C_max_ of placebo (766 ± 142.7 ng/mL) was lower than *Amaranthus*. The mean Cmax of product B (3472.56 ± 655.38 ng/mL), product C (3845.34 ± 6.96 ng/mL), and product D 3710.87 ± 410.94 ng/mL) were found to be similar. The placebo administration (0.72 ± 0.86 hour) showed significantly different T_max_ compared with the amaranth extracts. Placebo administration showed a higher elimination half-life (11.9 ± 4.66 hours), compared with the *Amaranthus* administrations with elimination half-life of 9.43 ± 3.49 hours, 7.72 ± 1.89 hours, and 8.4 ± 3.14 hours for products B, C, and D, respectively.

The linear model and pairwise *comparison* outcomes of log-transformed Cmax, AUC0-t, and AUC0-∞, when compared within the (−20%, 25%) range, indicated bioequivalence among the *Amaranthus* products A, B, and C. Conversely, the Cmax, AUC0-t, and AUC0-∞ values observed for the placebo did not demonstrate bioequivalence with the *Amaranthus* products for saliva nitrate (Table 5). The bioavailability of *Amaranthus* products A, B, and C is notably 1.6 times higher than that of the placebo.

**Table 5:** Bioequivalence assessment of different products for saliva nitrate.

Dependent variable			Mean difference (I–J)	SE	Sig.	95% CI		95% CI (Transformed)		
						lower bound	Upper bound	Lower bound	Upper bound	Bioequivalence
Cmax	A	B	−0.65829*	0.026	0.0000	−0.7279	−0.5887	−51.71	−44.49	False
		C	−0.70825*	0.026	0.0000	−0.7779	−0.6386	−54.06	−47.20	False
		D	−0.69209*	0.026	0.0000	−0.7617	−0.6225	−53.31	−46.34	False
	B	C	−0.04996	0.026	0.2410	−0.1196	0.0197	−11.27	1.99	True
		D	−0.0338	0.026	0.5770	−0.1034	0.0358	−9.83	3.65	True
	C	D	0.01615	0.026	0.9280	−0.0535	0.0858	−5.21	8.96	True
AUC0_t	A	B	−0.44463*	0.030	0.0000	−0.5228	−0.3664	−40.72	−30.68	False
		C	−0.51594*	0.030	0.0000	−0.5941	−0.4378	−44.80	−35.45	False
		D	−0.49492*	0.030	0.0000	−0.5731	−0.4167	−43.62	−34.08	False
	B	C	−0.07131	0.030	0.0860	−0.1495	0.0069	−13.89	0.69	True
		D	−0.05029	0.030	0.3330	−0.1285	0.0279	−12.06	2.83	True
	C	D	0.02102	0.030	0.8930	−0.0572	0.0992	−5.56	10.43	True
AUC0_inf	A	B	−0.40943*	0.025	0.0000	−0.4764	−0.3424	−37.90	−29.00	False
		C	−0.46293*	0.025	0.0000	−0.5299	−0.3959	−41.13	−32.70	False
		D	−0.45287*	0.025	0.0000	−0.5199	−0.3859	−40.54	−32.02	False
	B	C	−0.0535	0.025	0.1620	−0.1205	0.0135	−11.35	1.36	True
		D	−0.04344	0.025	0.3260	−0.1104	0.0235	−10.45	2.38	True
	C	D	0.01006	0.025	0.9790	−0.0569	0.077	−5.53	8.01	True

“*” represents statistically significant mean difference. Bioequivalence margins were set at 80%–125%.

### Analysis of saliva nitrite

**Figure 5:**
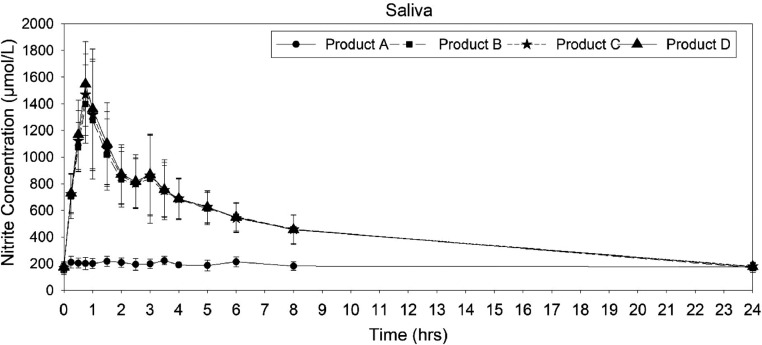
Mean nitrite saliva concentration−time curve after administration of placebo and *Amaranthus* products.

The mean saliva nitrite concentration−time profiles for nitrite found similar profiles for the *Amaranthus* products (products B, C, and D), whereas the saliva nitrite concentration profile of placebo (product A) was dissimilar to the *Amaranthus* products (Figure 5). The descriptive pharmacokinetic parameters are given in Table 2. The mean C_max_ of placebo (262.35 ± 10.49 ng/mL) was lesser than B, C, and D. The mean C_max_ of product B (1520.9 ± 302.6 ng/mL), product C (1681.2 ± 282.72 ng/mL), and product D (1585.83 ± 287.05 ng/mL) was found to be similar. The mean T_max_ was found comparable for the *Amaranthus* administration (0.79 ± 0.14 hours, 0.94 ± 0.57 hours, and 0.796 ± 0.14 hours for products B, C, and D, respectively). The T_max_ for the placebo administration (2.06 ± 1.48 hours) was significantly different compared with the amaranth products (Test statistic = 11.223, *P* = 0.011). A pairwise comparison result for T_max_ is given in **Supplemental Table 2**. Placebo administration showed a higher elimination half-life (115.98 ± 98.52 hours) compared with the *Amaranthus* administrations, with the elimination half-life of 11.13 ± 2.73 hours, 11.62 ± 2.56 hours, and 12.07 ± 2.88 hours for products B, C, and D, respectively (Table 5).

The linear model and pairwise comparison outcomes of log-transformed Cmax, AUC0-t, and AUC0-∞, when compared within the (−20%, 25%) range, indicated bioequivalence among the *Amaranthus* products A, B, and C. Conversely, the Cmax, AUC0-t, and AUC0-∞ values observed for the placebo did not demonstrate bioequivalence with the *Amaranthus* products for saliva nitrite (Table 6). The bioavailability of *Amaranthus* products A, B, and C is notably 2.5 times higher than that of the placebo.

**Table 6:** Bioequivalence assessment of different products for saliva nitrite.

Dependent variable			Mean difference (I–J)	SE	Sig.	95% CI		95% CI (Transformed)		
						Lower bound	Upper bound	Lower bound	Upper bound	Bioequivalence
Cmax	A	B	−0.75585*	0.02375	0.000	−0.8186	−0.6931	−55.90	−50.00	False
		C	−0.77529*	0.02375	0.000	−0.8380	−0.7125	−56.74	−50.96	False
		D	−0.80165*	0.02375	0.000	−0.8644	−0.7389	−57.87	−52.24	False
	B	C	−0.01943	0.02375	0.846	−0.0822	0.0433	−7.89	4.43	True
		D	−0.04580	0.02375	0.227	−0.1086	0.0170	−10.29	1.71	True
	C	D	−0.02636	0.02375	0.685	−0.0891	0.0364	−8.53	3.71	True
AUC0_t	A	B	−0.38255*	0.02117	0.000	−0.4385	−0.3266	−35.50	−27.86	False
		C	−0.38946*	0.02117	0.000	−0.4454	−0.3335	−35.94	−28.36	False
		D	−0.39154*	0.02117	0.000	−0.4475	−0.3356	−36.08	−28.51	False
	B	C	−0.00691	0.02117	0.988	−0.0629	0.0490	−6.09	5.03	True
		D	−0.00899	0.02117	0.974	−0.0649	0.0470	−6.29	4.81	True
	C	D	−0.00208	0.02117	1.000	−0.0580	0.0539	−5.64	5.54	True
AUC0_inf	A	B	0.31040*	0.06076	0.000	0.1498	0.4709	16.17	60.15	False
		C	0.29284*	0.06076	0.000	0.1323	0.4534	14.14	57.36	False
		D	.209624*	0.06076	0.000	0.1357	0.4568	14.53	57.90	False
	B	C	−0.01756	0.06076	0.992	−0.1781	0.1430	−16.31	15.37	True
		D	−0.01416	0.06076	0.995	−0.1747	0.1464	−16.03	15.77	True
	C	D	0.00340	0.06076	1.000	−0.1571	0.1640	−14.54	17.82	True

“*” represents statistically significant mean difference. Bioequivalence margins were set at 80%–125%.

In this study, nausea and vomiting tendency (mild) was reported by 1 subject in placebo and 1 in test, stomach cramps (mild) by 1 subject in test, diarrhea (moderate) by 2 subjects in test and 3 subjects in placebo, abdominal discomfort (mild) by 3 in test and 1 in placebo. The symptoms resolved themselves on the same day. There were no clinically significant changes in the biochemical or hematological parameters or no significant variabilities of vital variables evident during the course of study. The observations from this study highlight the efficient nitrate/nitrite absorption and conversion dynamics of *Amaranthus* species compared with the placebo. This provides a foundation for understanding the role of plant-based dietary supplements in contributing to NO bioavailability that can have beneficial implications on cardiovascular and neurologic health. Pharmacokinetic differences between *Amaranthus* species and placebo and observed bioavailability from this study is detailed in the following discussion section in comparison with existing literatures to explore their clinical and regulatory significance.

## Discussion

*Amaranthus* is a traditional edible vegetable with a good source of protein and nitrates. Considering the high nitrate content of *Amaranthus*, this study looked into the nitrate and nitrite concentration in blood and saliva among the human subjects administered with 2000 mg nitrate-rich extract of *Amaranthus*, in comparison with placebo administration. Saliva nitrate bioequivalence studies are important in pharmacokinetics, considering its pivotal role in enhancing systematic NO bioavailability through the enterosalivary circulation of nitrates. Salivary nitrate is secreted by salivary glands and reduced to nitrite by oral microbiota, facilitating a sustained controlled release of nitrite into systematic circulation, resulting in prolonged NO availability.^18^ Moreover, saliva is considered an important secondary matrix in nitrate pharmacokinetics, especially for supplements by regulatory agencies such as the FDA and EMA, making saliva bioequivalence a critical factor in product standardization and approval.^19^ Although there are significant health benefits of dietary nitrates and nitrites, particularly in cardiovascular and neurovascular function, the majority of prior research has focused on individual nitrate-rich foods. A comprehensive understanding of nitrate and nitrite bioavailability from different *Amaranthus* species is still lacking. By investigating 3 *Amaranthus* species extracts (*A. hybridus, A. hypochondriacus*, and *A. tricolor*), alongside a placebo control in healthy adult human subjects under fasting conditions, this study offers valuable insights into the absorption, distribution, metabolism, and excretion of these bioactive compounds. The obtained result implies a novel step toward the understanding of the characteristics of nitrate and nitrite pharmacokinetic properties originating from *Amaranthus* extracts.

One of the important observations from the present study is the significantly higher plasma and saliva nitrate and nitrite concentration−time profiles in the amaranth extracts (products A, B, and C) compared with the placebo. This variability clearly points to the distinct pharmacokinetic characteristics intrinsic to *Amaranthus* extracts with unique absorption mechanism and systematic exposure compared with the inert placebo.^20^ Furthermore, the observed variations in C_max_ (maximum plasma or saliva concentration), T_max_, and T_1/2_ between *Amaranthus* products and placebo further highlight the pharmacokinetic diversity among these extracts. The higher C_max_ values from *Amaranthus* products compared with placebo shows improved bioavailability and more efficient delivery of nitrates and nitrites into systemic circulation by amaranth products. Moreover, the consistent T_max_ values across the 3 *Amaranthus* products suggest rapid absorption kinetics and prompt onset of action compared with placebo. It is important to note that the shorter elimination half-life of *Amaranthus* extracts suggests faster clearance and reduced systemic exposure over time, which may have implications for dosing regimens and treatment efficacy.

It is important to note that the C_max_ values were similar for all 3 *Amaranthus* species extracts, suggesting no interspecies differences in delivering nitrates and nitrites into the body. Moreover, the time to reach maximum concentration (T_max_) values was similar for the *Amaranthus* products (∼1 hour), indicating rapid absorption and onset of action by the 3 species. Furthermore, the bioequivalence analysis revealed significant differences in C_max_, AUC_0-t_, and AUC_0-∞_ values between placebo and *Amaranthus* products (B, C, and D), affirming the superior bioavailability of nitrates and nitrites from *Amaranthus* formulations.

The reduced efficiency of NO generation and its pathway, especially from endothelial dysfunction, plays a major role in causing cardiovascular diseases. The important pathway for the endogenous NO production is by the oxidation of the guanidino nitrogen group of L-arginine by the NO synthase enzyme located in the vascular endothelium.^21^ For patients with severe endothelial dysfunction, there were efforts in providing L-arginine supplementation to improve NO production, but the inability to convert L-arginine to NO in those patients made the clinical trial fail.^22^ Similarly, people involved in severe physical activities also require extra NO to compete hypoxia. It is important to note that in this study, though the nitrate and nitrite concentration in plasma and saliva reached maximum concentration within 1 hour for both amaranth extracts and the placebo, the concentration of these compounds was significantly high among participants when administered with amaranth compared with the placebo. Because the nitrite and nitrate are 2 major metabolites of NO, the significant increase in nitrate and nitrite in plasma as well as saliva using the amaranth extracts indicate the enhanced NO level in the body. There were evident fluctuations in nitrite in the plasma, possibly from the conversion due to the continuous conversion of nitrite into nitrate with the help of facultative bacteria.^23^

Studies reported that continuous supplementation of nitrate and nitrite increases the overall NO content resulting in the potential reversal of endothelial NO deficiency from animal and human studies.^6^^,^^24^, ^25^, ^26^ Increased availability of NO could potentially improve cerebral blood flow and cognitive abilities. Because the brain ages, it experiences shrinkage and a reduction in its ability to produce ATP through oxidative phosphorylation.^27^ This decline, coupled with chronic white matter ischemia, contributes to cognitive decline. Additionally, age-related mitochondrial dysfunction has been linked to neuronal loss as seen in neurodegenerative diseases.^28^ Recent research indicates that NO plays a crucial role in cerebral vasodilation, neurotransmission, and the regulation of local cerebral blood flow in response to neural activity.^29^ Therefore, supplementing the diet with nitrate may hold promise in altering cerebrovascular function and improving cognitive performance. This study efficiently shows higher bioavailability of amaranth extract as a potential supplement for NO production so that age-related dysfunctions and cardiovascular damages can be controlled. The proved bioequivalence of the 3 amaranth extract species from this study aligns with the standards following the FDA and EMA bioequivalence guidelines, indicating that any of the 3 species could be used interchangeably in dietary formulations without significantly compromising nitrate/nitrite bioavailability. The outcomes from this study, hence, could support future regulatory approval of *Amaranthus*-based supplementation as dietary or therapeutic alternatives to synthetic nitrate/nitrite formulations.

Findings from this study underscore the potential therapeutic advantages of *Amaranthus*-based interventions in delivering NO precursors for cardiovascular health and other physiologic processes. Considering this ability to enhance the nitrate/nitrite level, *Amaranthus* extracts can be potentially used as a natural and dietary alternative to existing nitrate supplementation, after further studies to assess whether the observed effects can be significantly translated into clinically meaningful improvements in cardiovascular functioning. Sample size constraints from this study, interindividual variability in nitrate metabolism, role of external factors including hydration status, and circadian variation in NO metabolism all can influence the observed findings and act as a potential source of bias and limitations of this study. Future studies with control over these bias factors are warranted to improve the robustness of findings and to improve the generalizability of the research findings. The administration of *Amaranthus* products was well tolerated in this study, with no reported adverse effects or significant changes in biochemical or hematological parameters. This underscores the safety profile of *Amaranthus* extract formulations and supports their potential as novel therapeutic agents for various health conditions.

In conclusion, the findings of this study contribute to our understanding of the pharmacokinetic properties of *Amaranthus* extract formulations and their potential clinical applications. Moving forward, it is important to have long-term clinical trials to assess the sustained effects of *Amaranthus*-based supplementation on cardiovascular health, cognitive function, etc. The assessment of optimal dosing regimens of *Amaranthus*-based supplements and their effect on other dietary interventions could help refine the therapeutic benefits of *Amaranthus* extracts.

## Conclusion

This 4-way placebo-controlled bioavailability study of nitrate and nitrites in 3 different species of *Amaranthus* found that the 3 species *A. hybridus, A. hypochondriacus*, and *A. tricolor* are bioequivalent in terms of plasma and saliva nitrate and nitrite availability and evidently have higher bioavailability than placebo. The findings clearly reported that 2000 mg of extract leads to elevated levels of nitrate and nitrite in the body, compared with placebo, and suggest that *Amaranthus*-based supplements could be used as a potential natural alternative to synthetic nitrate supplements. However, this study warrants further clinical investigations on long-term efficiency before generalization. Although this study clearly confirms the bioavailability of *Amaranthus* extracts, the limitations identified in this study need to be addressed in future studies considering long-term effects, optimal dosing regimens, mechanistic pathways, and the role of external factors.

## Funding

This research received no specific grant from any funding agency in the public, commercial, or not-for-profit sectors.

## Declaration of competing interest

The authors have no conflicts of interest to declare.

## References

[1] Ashworth A., Mitchell K., Blackwell J.R., Vanhatalo A., Jones AM. (2015). High-nitrate vegetable diet increases plasma nitrate and nitrite concentrations and reduces blood pressure in healthy women. Public Health Nutr.

[2] Ranasinghe R., Marapana R. (2018). Nitrate and nitrite content of vegetables: a review. J Pharmacogn Phytochem.

[3] Venskutonis P.R., Kraujalis P. (2013). Nutritional components of amaranth seeds and vegetables: a review on composition, properties, and uses. Compr Rev Food Sci Food Saf.

[4] Bondonno C.P., Croft K.D., Hodgson JM. (2016). Dietary nitrate, nitric oxide, and cardiovascular health. Crit Rev Food Sci Nutr.

[5] Machha A., Schechter AN. (2011). Dietary nitrite and nitrate: a review of potential mechanisms of cardiovascular benefits. Eur J Nutr.

[6] Omar S., Webb A., Lundberg J., Weitzberg E. (2016). Therapeutic effects of inorganic nitrate and nitrite in cardiovascular and metabolic diseases. J Intern Med.

[7] Stanhewicz A.E., Kenney WL. (2017). Role of folic acid in nitric oxide bioavailability and vascular endothelial function. Nutrition Rev.

[8] Medina-Leyte D.J., Zepeda-García O., Domínguez-Pérez M., González-Garrido A., Villarreal-Molina T., Jacobo-Albavera L. (2021). Endothelial dysfunction, inflammation and coronary artery disease: potential biomarkers and promising therapeutical approaches. Int J Mol Sci.

[9] Stokes K.Y., Dugas T.R., Tang Y., Garg H., Guidry E., Bryan NS. (2009). Dietary nitrite prevents hypercholesterolemic microvascular inflammation and reverses endothelial dysfunction. Am J Physiol Heart Circ Physiol.

[10] Toda N., Ayajiki K., Okamura T. (2009). Cerebral blood flow regulation by nitric oxide: recent advances. Pharmacol Rev.

[11] Lu B., Nagappan G., Lu Y. (2014). BDNF and synaptic plasticity, cognitive function, and dysfunction. Handb Exp Pharmacol.

[12] Nyonje WA. Nutrients, anti-nutrients and phytochemical evaluation of ten vegetable amaranth (Amaranthus spp.) varieties at two stages of growth. 2015.

[13] Rastogi A., Shukla S. (2013). Amaranth: a new millennium crop of nutraceutical values. Crit Rev Food Sci Nutr.

[14] Kapil V., Milsom A.B., Okorie M. (2010). Inorganic nitrate supplementation lowers blood pressure in humans: role for nitrite-derived NO. Hypertension.

[15] Han S.S., Nam E.C., Won J.Y. (2012). Clonazepam quiets tinnitus: a randomised crossover study with Ginkgo biloba. J Neurol Neurosurg Psychiatry.

[16] Bose M., Dey A. (2015). Crossover designs. Handbook of Design and Analysis of Experiments.

[17] Chen M.L., Shah V., Patnaik R. (2001). Bioavailability and bioequivalence: an FDA regulatory overview. Pharm Res.

[18] Lundberg J.O., Weitzberg E., Cole J.A., Benjamin N. (2004). Nitrate, bacteria and human health. Nat Rev Microbiol.

[19] Idkaidek NM. (2017). Comparative assessment of saliva and plasma for drug bioavailability and bioequivalence studies in humans. Saudi Pharm J.

[20] Subramanian D., Gupta S. (2016). Pharmacokinetic study of amaranth extract in healthy humans: a randomized trial. Nutrition.

[21] Moncada S., Higgs E., Hodson H. (1991). The L-arginine: nitric oxide pathway. J Cardiovasc Pharmacol.

[22] Wilson A.M., Harada R., Nair N., Balasubramanian N., Cooke JP. (2007). L-arginine supplementation in peripheral arterial disease: no benefit and possible harm. Circulation.

[23] Ding L., Han B., Zhou J. (2022). Characterization of the facultative anaerobic Pseudomonas stutzeri strain HK13 to achieve efficient nitrate and nitrite removal. Process Biochem.

[24] Hobbs D.A., George T.W., Lovegrove JA. (2013). The effects of dietary nitrate on blood pressure and endothelial function: a review of human intervention studies. Nutr Res Rev.

[25] Kiani A.K., Bonetti G., Medori M.C. (2022). Dietary supplements for improving nitric-oxide synthesis. J Prev Med Hyg.

[26] Zand J., Lanza F., Garg H.K., Bryan NS. (2011). All-natural nitrite and nitrate containing dietary supplement promotes nitric oxide production and reduces triglycerides in humans. Nutr Res.

[27] Cuenoud B., Ipek Ö, Shevlyakova M. (2020). Brain NAD is associated with ATP energy production and membrane phospholipid turnover in humans. Front Aging Neurosci.

[28] Elfawy H.A., Das B. (2019). Crosstalk between mitochondrial dysfunction, oxidative stress, and age related neurodegenerative disease: etiologies and therapeutic strategies. Life Sci.

[29] Lourenço C.F., Laranjinha J. (2021). Nitric oxide pathways in neurovascular coupling under normal and stress conditions in the brain: strategies to rescue aberrant coupling and improve cerebral blood flow. Front Physiol.

